# Supplementary material for: Songbird parents coordinate offspring provisioning at fine spatio‐temporal scales
Source: J Anim Ecol. 2022 Apr 27;91(6):1316–26. doi: 10.1111/1365-2656.13702 (PMC9321892; doi:10.1111/1365-2656.13702)
Supplement: Supplementary file 1 — Appendix S1 [file JANE-91-1316-s001.pdf]

**Supplementary material**

***S1 - Validation of triangulation algorithm***

The Encounternet triangulation algorithm uses the RSSI (received signal strength indication) values recorded by the receivers. The RSSIs values decreases non-linearly with the distance between the tag and receivers, as well as the probability of the receivers to log a tag pulse (Figure S1A-B)(Rutz et al., 2015). The algorithm then uses the RSSIs to estimate the distance between the tag and each receiver by using the following linear RSSI-to-distance conversion function was:

*distance* (in m) = 4 – 0.2 RSSI (green line in Figure S1A).

The estimated distances to each receiver are subsequently used in a Multi-Dimensional Scaling analysis (Cox and Cox, 2008; Davison, 1983) (Matlab function "mdscale") to generate the tag location coordinates that best match the distances to each base station. Intuitively, the adopted linear conversion function (green line in Figure S1A) underestimates and equalizes the real distances between the receivers and the tag (*e.g.* receivers logging RSSIs of 20, 0 and -20 are estimated to respectively be at 0, 4 and 8m from the tag), such that the Multi-Dimensional Scaling analysis tends to estimate the tag locations at the centre of the detection area (portion of space in which the inside receivers detect the signal). Conversely, the use of empirical non-linear RSSI-to-distance conversion functions (*e.g.* red line in Figure S1A), due to the high vertical variance in figure S1A, would tend to highly overestimate a great majority of the receiver-tag distances (*i.e* the datapoints underneath the red line in Figure S1A; for instance a receiver logging an RSSI of -20 and located at 25m from the tag would be estimated at ca150m from the tag), determining the estimated tag locations to fall outside the array.

To validate the Encounternet triangulation algorithm, in autumn 2014 we carried out a field test with the Encounternet tracking system in a forest of mixed wood located at Westerheide, The Netherlands (52°01'N, 5°85'E). In two different areas within the field site, we built an array of 37 receivers in a 75-m array with the same configuration of the arrays deployed during the field season in 2016 (see Methods section). Within each array, we randomly selected 40 tag sites distributed at different distances (ranging from to 4.9 to 72.8m) from the centre of the array. The coordinates of the receivers as well as those of the tag sites were located and surveyed in the field with a survey-grade GPS (Ashtech ProMark 800, Santa Clara, CA, U.S.A.). Each tag site was visited for two minutes with two active radio-tags mounted on a plastic support at the top of a 4m extending glass fiber pole. The tags could be oriented in six different orientations: the antenna could point upwards, downwards, towards the centre of the array, towards the

border of the array, or positioned horizontally with a 90- or 270-degree angle from the centre of the array). The orientation of the two tags differed from one another in each tag site and was randomized across tag sites.

For each tag in each tag site, we estimated the locations of the tag from one pulse randomly selected within the two-minute intervals. The triangulation algorithm correctly estimated the angle of the tag locations relative to the centre of the array as the estimated angle of the tag sites strongly correlated with the actual angle [r= 0.91, $P < 0.001$; calculated with the function cor.circular in the R package 'circular' (Agostinelli and Lund, 2017)]. However, the accuracy in locating the tag (mean accuracy (mean ± SE): 20.69 ± 0.80) decreases as the distance between tag location and centre of the array increases ($F_{1,157.01} = 322.71$, $P < 0.001$). This effect was created by the triangulation algorithm which biases the estimated locations towards the centre of the array ($F_{1,157.91} = 16.30$, $P < 0.001$) so that locations at the edge of the array were estimated more inside the array (Figure S2A). This bias is likely to compress the parental utilization distributions (UDs) towards the centre of the array and to underestimate differences in UDs in the analysis (see Methods section). As a result, conclusions regarding differences in EMDs are conservative.

To assess the impact of this triangulation bias on the EMD analysis, we corrected the estimated distance of the tag from the centre of the array by adding the bias, estimated from the regression line of bias and estimated distance to the centre (red line in Figure S2B). With this correction, we improved the accuracy of the triangulation algorithm (accuracy of the algorithm after correction (mean ± SE): 13.62 ± 0.54 m), and we eliminated the effect of the original bias (the accuracy does no longer depend on the distance from the centre of the array: $F_{1,157.06} = 1.81$, $P = 0.179$; no bias toward the centre: $F_{1,158} = 0$, $P = 0.417$, Figure S2C). The accuracy of the estimated locations did not differ between the tags ($F_{1,152} = 0.19$, $P = 0.66$) and did not depend on their orientation ($F_{5,149.30} = 1.88$, $P = 0.10$). The UDs and EMD analyses (i.e. testing whether earth mover's distance between male and female UDs differed between lags) calculated with the corrected version of the triangulation algorithm did not alter the results and biological conclusion of the broad scale ($F_{14,170} = 3.24$, $P < 0.001$ Figure S3A) and fine scale analysis ($F_{10,1353} = 23.36$, $P < 0.001$, Figure S3B).

***S2 – Undetected locations.***

The Encounternet radio-tracking detected 73% of the parental locations (mean ± SE: 0.73 ± 0.05, range: 0.98 – 0.30) during the 64 hours of data collection so that the UDs used in the analyses represent a portion of their real unknown UDs. These undetected locations could occur either because parents

moved outside the array and its detection area, or because parents were inside the array but in areas of difficult signal propagation, e.g., on the floor. To tease apart these two alternatives, in the 2014 field tests we also placed the tags on the ground and at eight meter of the ground for two minutes in 23 of the 40 tag sites. The probability of detecting and triangulating the locations of tags on the ground was significantly lower than at four and eight meters ($z$ = 11.48, $P$ < 0.001; $z$ = 11.03, $P$ < 0.001 respectively) with a probability of detection equal to 81% ± 4 (mean ± SE), 99% ± 0 and 99% ± 0 for tags on the ground, at four and eight meters, respectively (Figure S4). This indicate that a portion of the missing locations might be due to birds foraging on the ground albeit this behaviour seems rare (*DB personal observations on provisioning great tits*). To understand whether periods of missing locations occurred due to birds leaving the array to forage further away from the nest we looked at the estimated distance from the nest of the last location before the birds were no longer detected. The average distance of the last estimated location was significantly greater than the average distance of the estimated locations (respectively 44.0m ± 0.42 and 22.2m ± 0.06; $t_{1291}$ = 50.41, $P$ < 0.001; Figure S5), suggesting that birds probably left the array in those occasions. In addition, the angle of the last estimated location relative to the nest position highly correlated with the angle of the first estimated location when the birds were located again (r= 0.82, $P$ < 0.001; Figure S6). Given these results, we assume that the presence of non-estimated locations occurred mainly due to practical equipment limitation in extending the array further away from the nest in all directions.

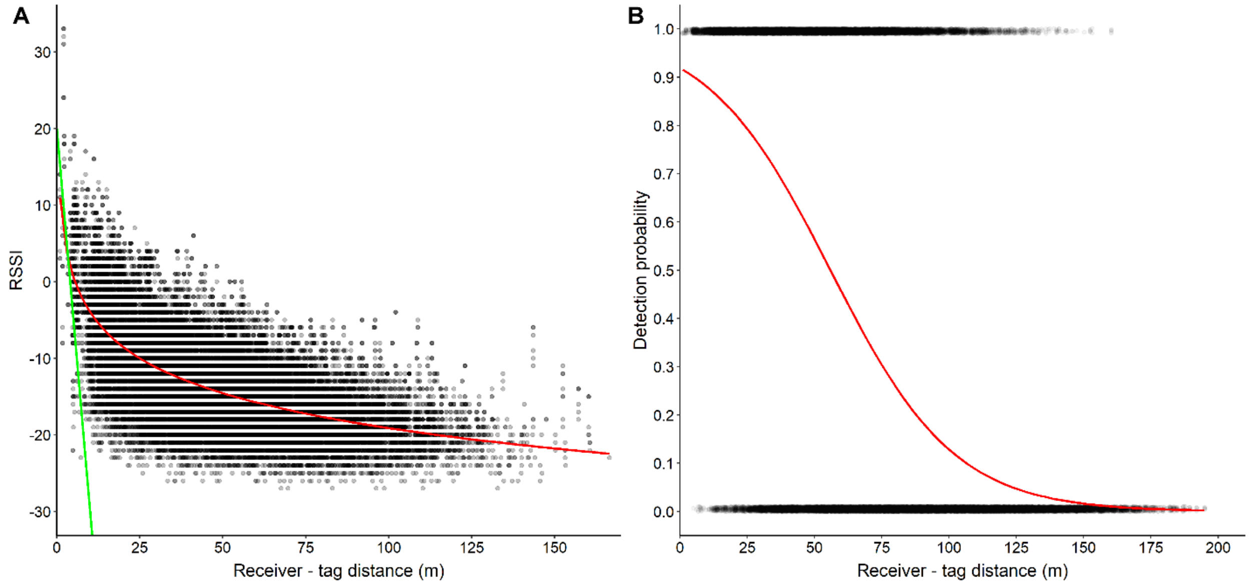


**Figure S1.** Empirical relationships for the RSSI against the receiver-tag distance (**A**); and the probability of a receiver to detect a signal against the receiver-tag distance (**B**). Data were collected during the field test in 2014.

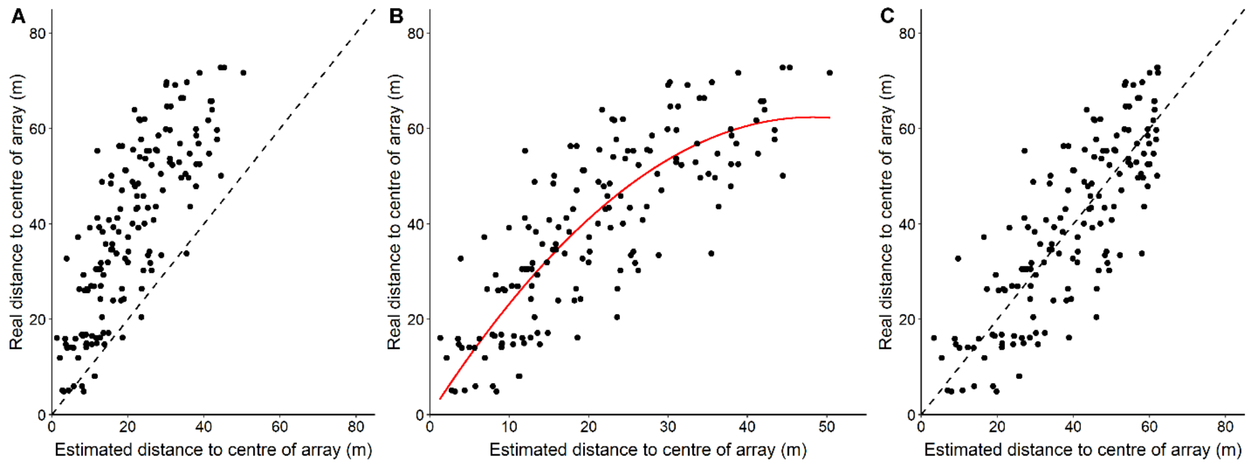


**Figure S2.** Relationship between real and estimated distance to the centre of the array for 160 locations before (**A, B**) and after (**C**) the correction of the triangulation algorithm. Red line in (**B**) represents the regression line used for the correction.

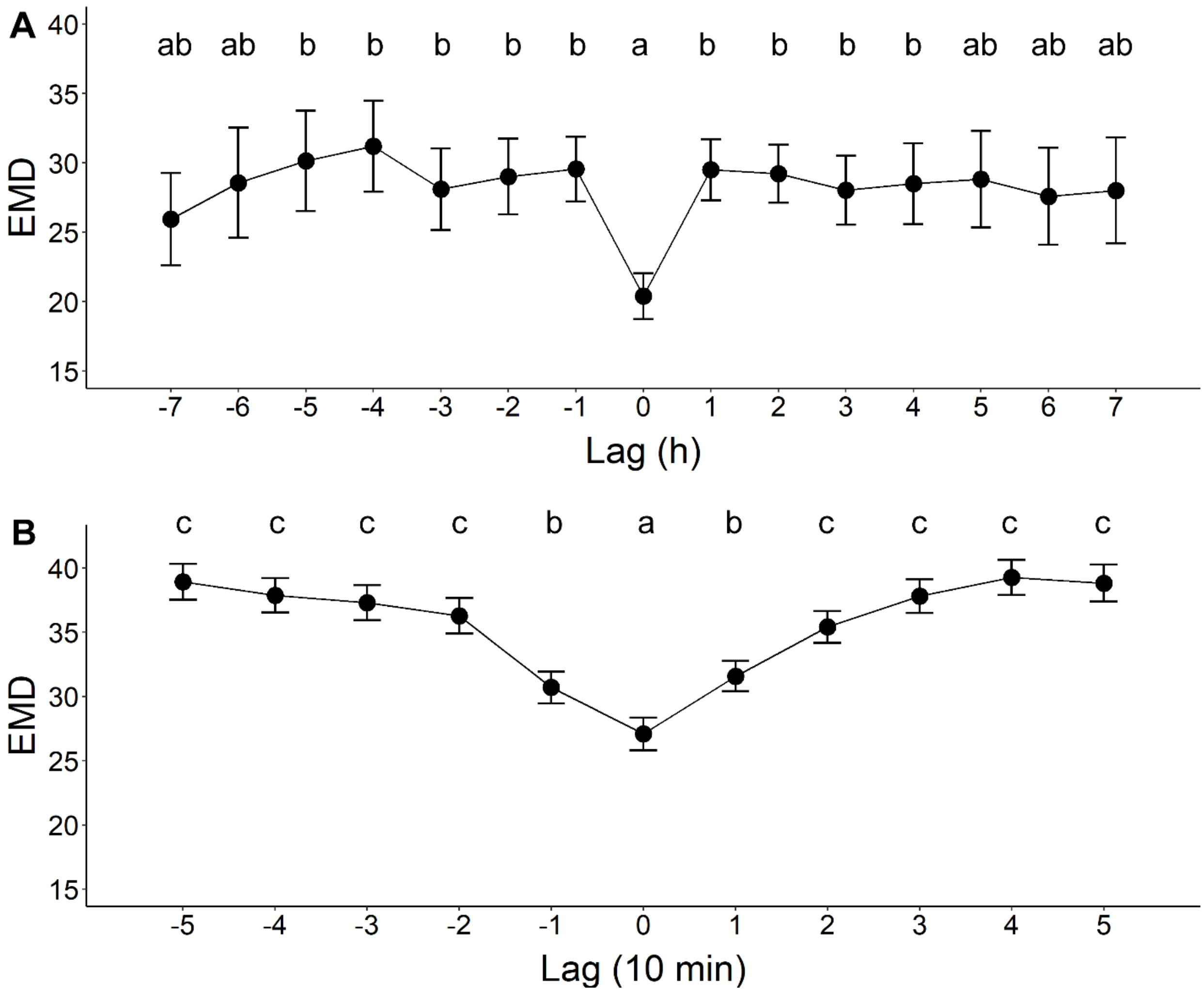


**Figure S3.** Broad (**A**) and fine (**B**) scale analysis when applying a correction to the Encounternet triangulation algorithm. Mean ± SE are given. Different letters indicate significant differences among lag classes in the post-hoc tests. Both figures show a pattern very similar to the results obtained by not applying the correction (Figures 2A and B)

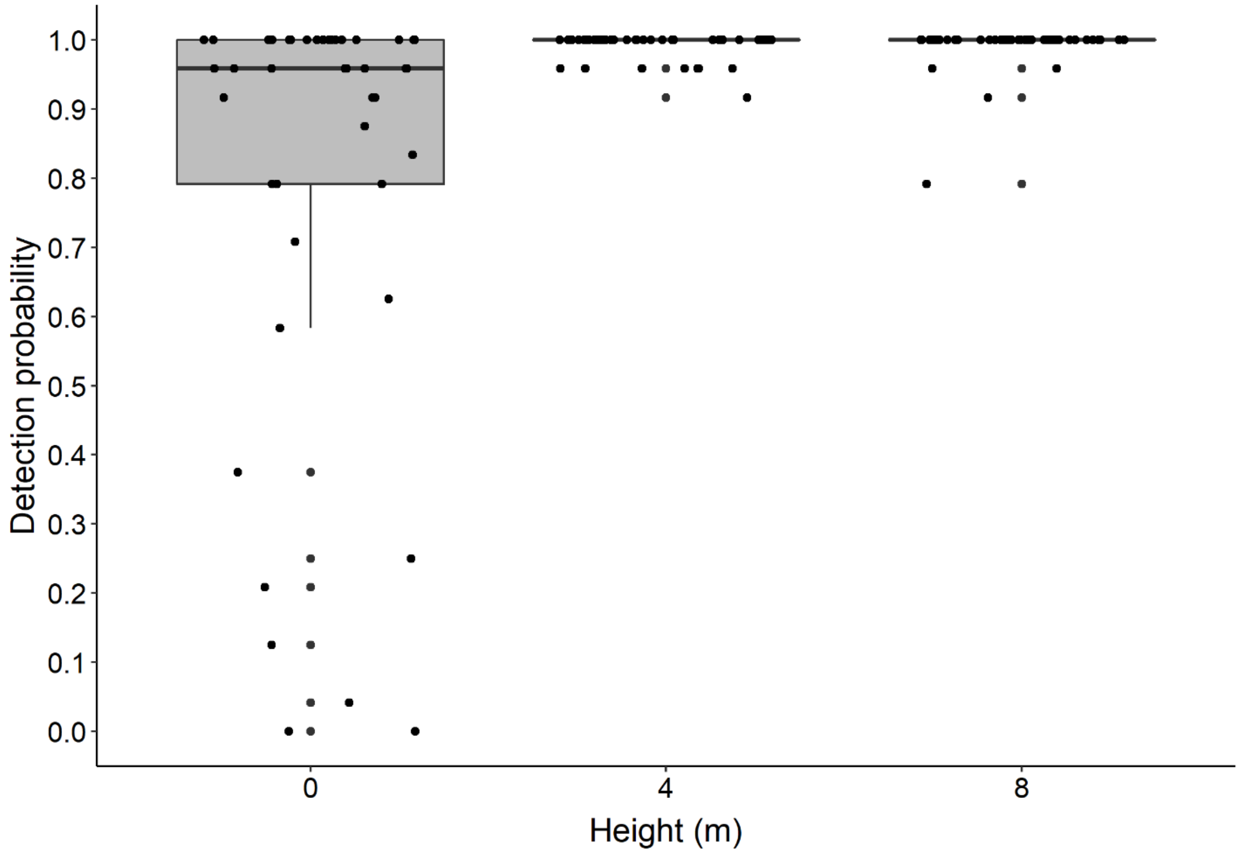


**Figure S4**. Boxplot of the probability of detecting and locating tags at different heights off the ground (0-4-8m respectively).

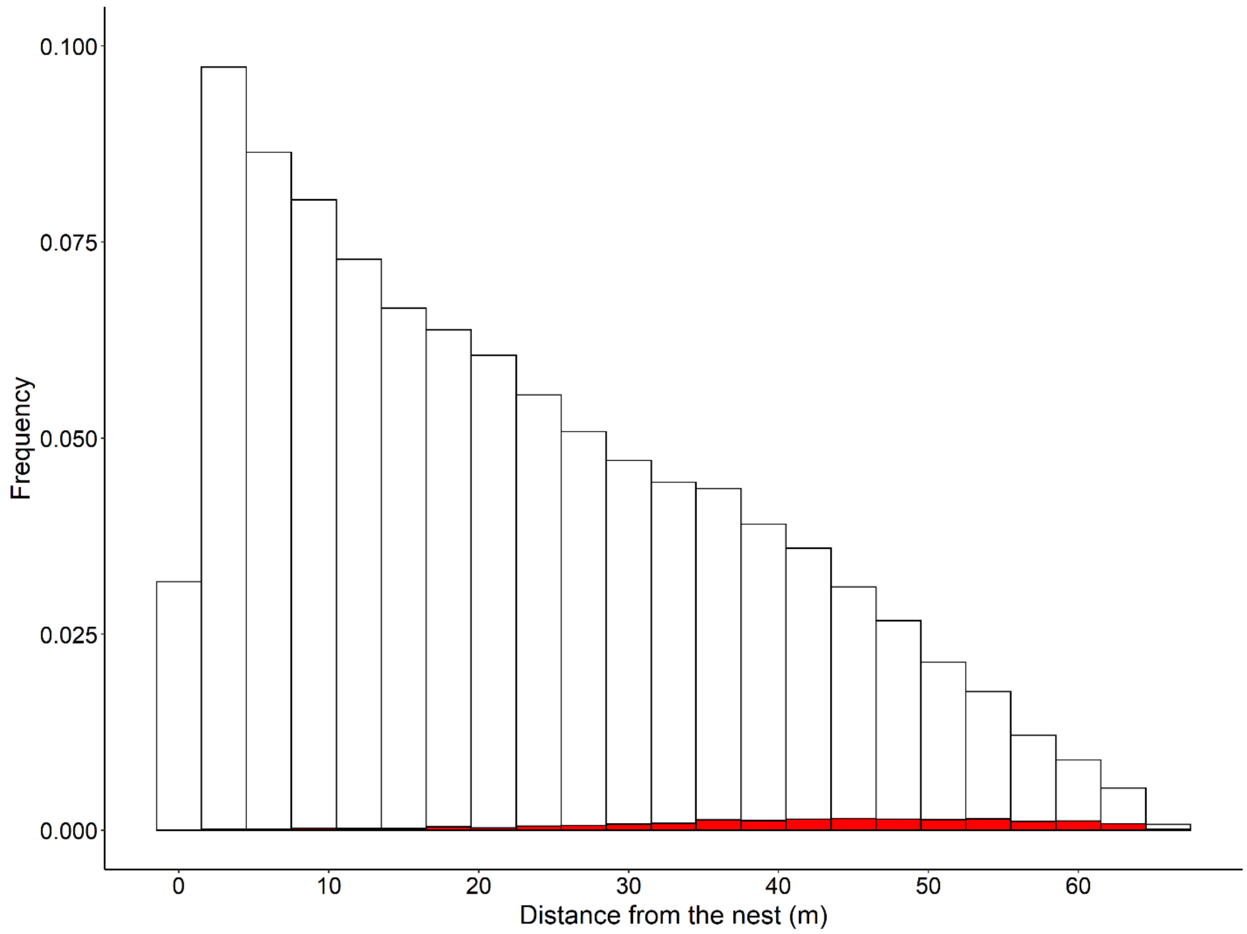


**Figure S5.** Frequency histogram of the distance from the nest of the estimated locations of great tit provisioning parents. The locations in red represent the last locations before the birds were not detected.

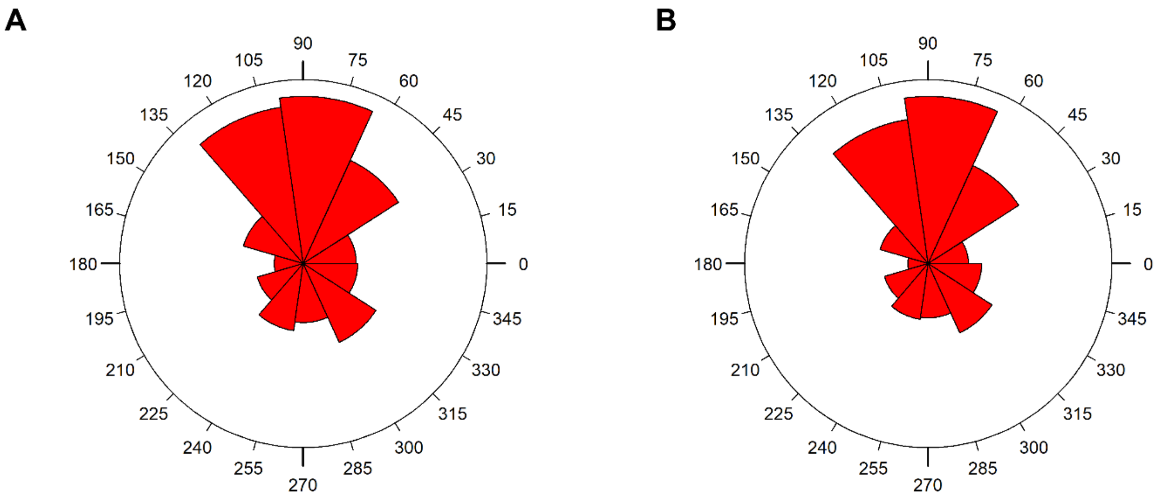


**Figure S6.** Histogram of the angle of the last locations before (**A**) and the first locations after (**B**) the birds' position were not detected.

**Cox, M. A. A. and Cox, T. F.** (2008). Multidimensional Scaling. In *Handbook of Data Visualization*, pp. 315-347. Berlin, Heidelberg: Springer Berlin Heidelberg.

**Davison, M. L.** (1983). Introduction to Multidimensional Scaling and Its Applications. *Applied Psychological Measurement* **7**, 373-379.

**Rutz, C., Morrissey, M. B., Burns, Z. T., Burt, J., Otis, B., St Clair, J. J. H. and James, R.** (2015). Calibrating animal-borne proximity loggers. *Methods in Ecology and Evolution* **6**, 656-667.
